# Outcomes after nonoperatively treated non-displaced Lisfranc injury: a retrospective case series of 55 patients

**DOI:** 10.1007/s00402-020-03599-w

**Published:** 2020-09-22

**Authors:** Ville T. Ponkilainen, Nikke Partio, Essi E. Salonen, Heikki-Jussi Laine, Heikki M. Mäenpää, Ville M. Mattila, Heidi H. Haapasalo

**Affiliations:** 1 Department of Orthopaedics, Faculty of Medicine and Life Sciences and Tampere University Hospital, University of Tampere, Teiskontie 35, PL2000, 33521 Tampere, Finland; 2 Pohjola Sairaala, Kelloportinkatu 1, 33100 Tampere, Finland; 3 COXA Hospital for Joint Replacement, Biokatu 6, 33520 Tampere, Finland; 4 University of Tampere, School of Medicine, 33520 Tampere, Finland

**Keywords:** Lisfranc, Injury, Tarsometatarsal, Nonoperative, Conservative, Treatment, Outcomes

## Abstract

**Background:**

Current knowledge of the role of the nonoperative treatment of Lisfranc injuries is based on a few retrospective case series. Hence, consensus on which patients can be treated nonoperatively does not exist. The aim of this study was to investigate outcomes after nonoperative treatment of Lisfranc injuries.

**Methods:**

In this study, patients were collected by recruiting all computer tomography-confirmed Lisfranc injuries treated during a 5-year period at a major trauma hospital. Between 2 and 6 years after suffering the injury, patients completed the visual analogue scale foot and ankle questionnaire.

**Results:**

In total, 55 patients returned adequately completed questionnaires and were included in the study. Of those, 22 patients had avulsion fractures and 33 had simple non-displaced intra-articular fractures. Of these patients, 30 (55%) scored over 90 points in both the pain and function subscales of the VAS-FA, and 35 (64%) scored over 90 points overall. In addition, three (5%) patients scored under 60 points in both the pain and function subscales of the VAS-FA, and four (7%) scored under 60 points overall. Only one patient with avulsion fractures underwent secondary surgery.

**Conclusion:**

Nonoperative treatment has a role in the treatment of Lisfranc injuries, and the results of our study support the view that avulsion and simple intra-articular fractures with < 2 mm of displacement can be treated nonoperatively with high functional outcomes. The results of nonoperative and operative treatment should be compared in a prospective randomized controlled study setting in future studies.

**Level of evidence:**

IV, retrospective case series

## Introduction

The ‘Lisfranc injury’ comprises a broad spectrum of tarsometatarsal (TMT) joint injuries that range from subtle injuries to complete dislocation [13, 20, 22, 28]. The incidence of these injuries has been found to be more common (9.2/100,000/person years) than previously thought, and more subtle injuries are found nowadays due to more precise diagnostics [25].

The first studies investigating Lisfranc fracture dislocations, published before 1990, consecutively recommended operative treatment for Lisfranc injuries, since nonoperative treatment led to unfavorable outcomes [1, 3, 6, 13]. At the time, however, nonoperative treatment was performed using closed reduction, lacked adequate aftercare [1, 3, 6], or the reported injuries were primarily missed [13]. More recently, only a few retrospective case series have been published that investigate the nonoperative treatment of Lisfranc injuries. These studies have had several limitations [4, 5, 22, 34]. In all previous studies, the diagnosis of the injury has been based on plain radiographs, and the outcomes evaluated without valid measures [4, 5, 8, 22, 34]. Nevertheless, more recent studies have focused purely on the operative techniques, disregarding the nonoperative treatment option almost completely [14, 16, 17].

Based on the findings of previous retrospective studies, a displacement of 2 mm or more between the medial cuneiform and the base of the second metatarsal bone is considered to be a sign of instability. Furthermore, it has been recommended that these patients are treated operatively to achieve higher functional outcomes and to lower the risk of post-traumatic osteoarthritis [2, 5, 7, 8, 11, 13, 16, 19, 23, 24, 29, 30, 34]. To date, only two randomized controlled trials (RCT) have investigated the treatment of Lisfranc injuries by comparing open reduction and internal fixation (ORIF) and primary arthrodesis (PA) [14, 17]. Despite the high-quality study methods, the preoperative diagnosis in these trials was based on plain radiographs, and heterogenous and not properly validated outcome measures were used [14, 17]. Interestingly, to date, there have been no RCTs that compare the nonoperative and operative treatment of Lisfranc injuries, and thus the current knowledge on the role of nonoperative treatment is based on a few retrospective case series [4, 5, 8, 34]. Hence, consensus on which patients can be treated nonoperatively does not at present exist. The aim of this retrospective study is, therefore, to investigate the outcomes after nonoperative treatment of Lisfranc injuries.

## Materials and methods

This study was conducted at a major trauma hospital, serving a catchment area of a half-million residents. Patients included in this study were collected by reviewing all CT studies (traditional CT or cone beam CT [CBCT]) that had been performed due to an acute injury to the foot and ankle region during a 5-year period (1.1.2012 to 31.12.2016). CBCT imaging of the foot was performed with Planmed Verity extremity CT (Planmed Oy, Helsinki Finland) with a limited field of view (FOV) of 12 cm and a slice thickness of 0.2 mm. Image data were analyzed using a GE AW Server workstation and 1 mm true axial, sagittal, and coronal reformates, and three-dimensional (3D) volume rendering reformates were obtained. CT imaging of the foot was performed with either a 64-slice or a 128-slice CT scanner with 0.5–0.63 mm slice thickness and both bone and soft tissue rendering was used. Further, similar post-processing for two-dimensional (2D) and 3D reformates was performed.

**Fig. 1:**
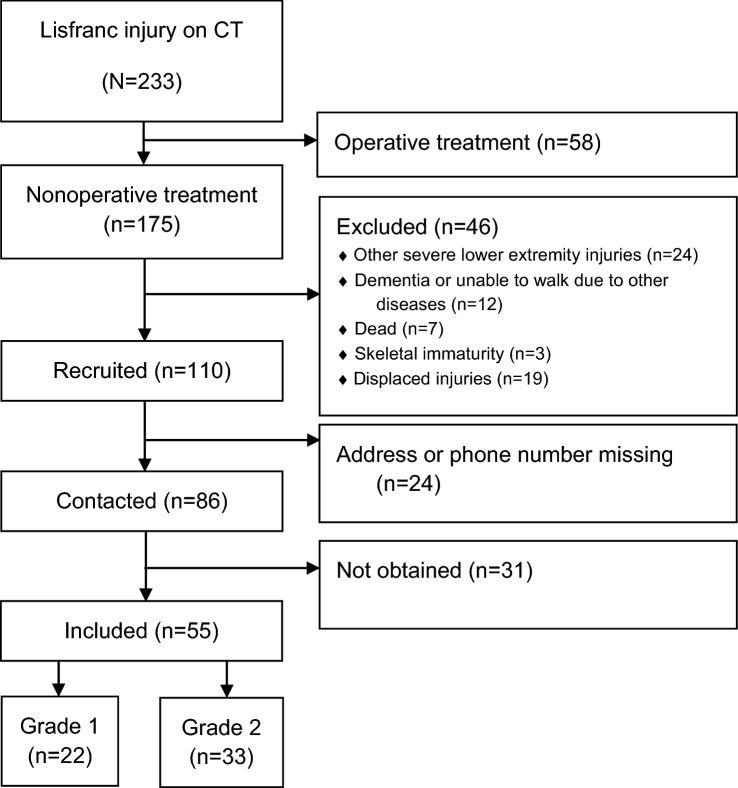
Flowchart of the study

All patients who were initially treated due to a Lisfranc injury were included in the study. The clinical characteristics of the patients were collected from medical records retrospectively. Patients were contacted by mail between 2 and 6 years after the injury and recruited to participate in this study. The recruited patients provided a written consent form for their participation. In total, 233 patients with Lisfranc injuries were identified. Of these, 175 (75%) were treated nonoperatively and 58 (25%) operatively. The patients competed a foot- and ankle-specific patient-reported outcome measure (PROM): the visual analogue scale foot and ankle (VAS-FA) [32]. The VAS-FA has been previously translated and validated into Finnish [31]. Of the contacted nonoperatively treated patients, 55 (64%) returned adequately completed questionnaires (Fig. 1). The study protocol was approved by the Ethics Committee of Pirkanmaa Hospital District.

### Nonoperative treatment protocol

Our hospital’s current treatment policy for non-displaced Lisfranc injuries is that all patients with < 2 mm of dislocation in the second and third tarsometatarsal joints or between the second metatarsal and the medial cuneiform in non-weightbearing CT imaging will be treated nonoperatively. The standard nonoperative treatment of a non-displaced Lisfranc injury in our hospital has been usually conducted with non-weightbearing immobilization in a cast for 4–6 weeks followed by progressive weightbearing towards full weightbearing during the next 4 weeks.

### Classification

For this study, the patients were divided into three grades by modifying the CT-based classification by Schepers and Rammelt [33]. The strengths of this classification are that it is CT based and it approaches the Lisfranc joint from the columnar perspective [33]. Since the previous commonly used classifications have focused on either severe fracture dislocations [13, 20] or on the diastasis between the first and the second TMT joints [22], it is important that this more recent classification takes into account the whole joint and the whole range of injuries. The Schepers and Rammelt classification does, however, have few drawbacks. For example, it classifies each column (medial, central, or lateral) and type of the injury separately (ligamentous, simple, or comminuted), resulting in dozens of different classes that make the everyday clinical use of the classification difficult. In addition, it does not take into account the displacement, even though this might be an important factor, especially when choosing between nonoperative and operative treatment. To further simplify the classification, we divided the patients into three different grades: 1—ligamentous injuries with avulsions, 2—simple intra-articular fractures, and 3—comminuted or more than 2 mm dislocated fractures. Our hospital’s current treatment policy for grade 3 injuries is operative. Hence, those patients with grade 3 injuries were excluded from this study, as this group consisted of either patients that we were noncompliant, had refused operative treatment or surgery was otherwise contraindicated.

### Statistical methods

Clinical and sosiodemographic data are presented as means with standard deviations (SD), medians with interquartile range (IQR), or as counts with percentages. Statistical analyses were performed using R (version 3.6.2).

## Results

**Fig. 2:**
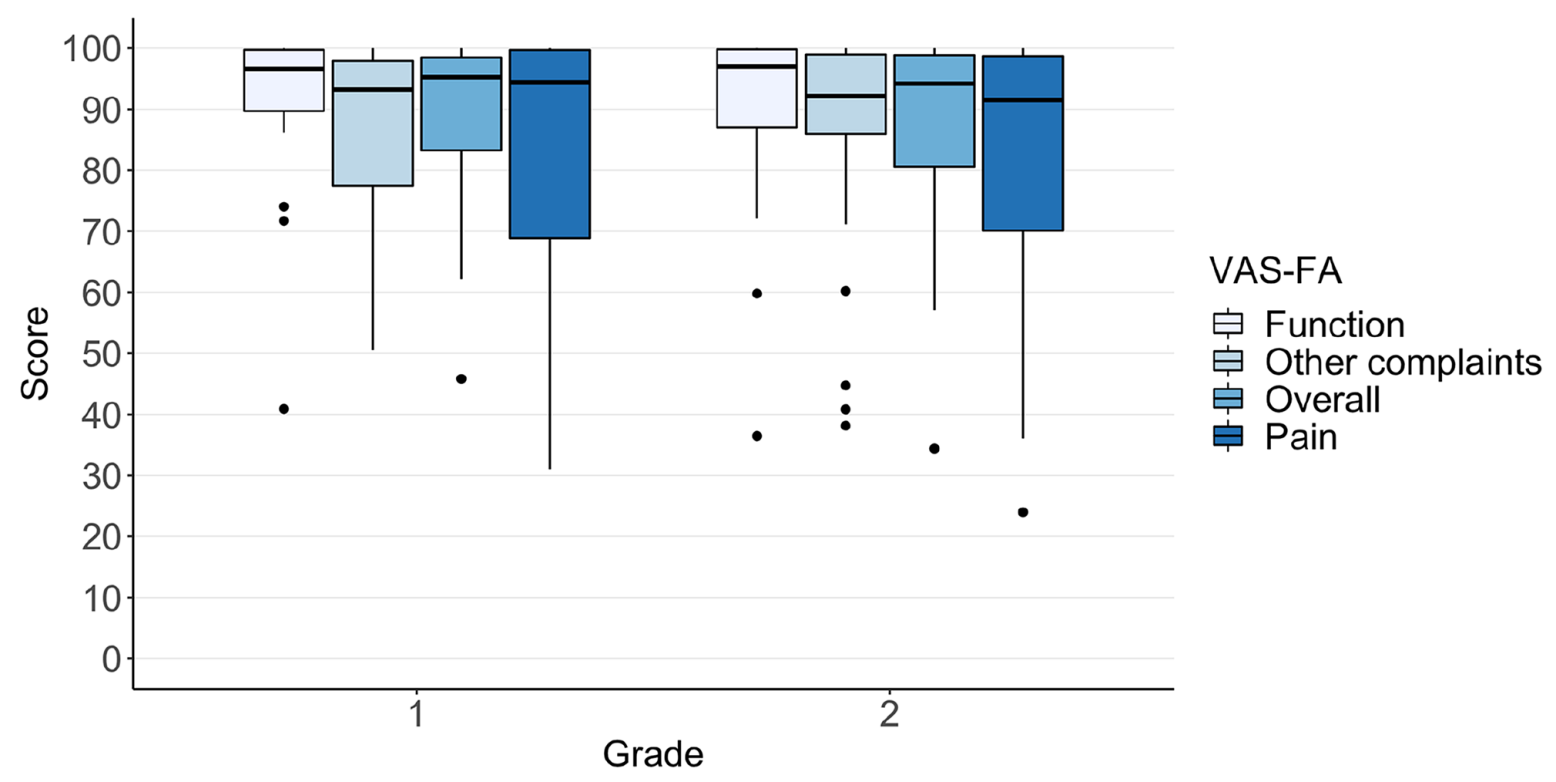
The visual analogue scale foot and ankle overall, pain, function and other complaints scores of the patients with nonoperatively treated Lisfranc injuries. Grade 1: ligamentous injuries with avulsions. Grade 2: simple intra-articular fractures

The characteristics of the 55 nonoperatively treated patients are presented in Table 1. Altogether, 48 (87%) of the patients were immobilized with a non-weightbearing cast for at least 4 weeks, and 39 (71%) of the patients for at least 6 weeks. A small number of the patients (*n* = 7) underwent shorter than the standard nonoperative treatment. After removing the cast, weightbearing was started as tolerated with a walking orthosis boot or normal shoe. The median VAS-FA scores of all patients were 95.1, 94.3, 97.0, and 92.2 for overall, pain, function, and other complaints, respectively. Out of all the patients, 30 (55%) scored over 90 points in both the Pain and Function subscales of the VAS-FA, and 35 (64%) scored over 90 points overall (Fig. 2). In total, 36 (65%) patients scored over 80 points in both the pain and function subscales of the VAS-FA, and 43 (78%) scored over 80 points overall. Of all the patients, three (5%) scored under 60 points in both the pain and function subscales of the VAS-FA, and four (7%) scored under 60 points overall.

**Table 1:** Background and clinical characteristics of the participants

	All^a^ (*N* = 175)	Included^b^ (*n* = 55)
Age, mean (SD)	38 (18)	42 (18)
Male, *n* (%)	124 (71)	33 (60)
Follow-up (years), median (range)	–	3.8 (2–6.6)
Time from injury to CT (days), median (range)	1 (0–29)	1 (0–29)
Trauma mechanism, *n* (%)		
Tumbling or twisting	63 (36)	19 (35)
Crush injury	32 (18)	13 (24)
Sports	12 (7)	6 (11)
Falling on stairs	12 (7)	2 (4)
Falling	17 (10)	5 (9)
Motor vehicle collisions	21 (12)	3 (5)
Bicycle collisions	7 (4)	3 (5)
Other	11 (6)	4 (7)

^a^All nonoperatively treated patients

^b^All nonoperatively treated non-displaced patients who adequately completed the questionnaires

Grade 1 injuries with avulsion fragments around TMT joints were found from 22 patients. Patients had injuries affecting from 1 to 3 TMT joints (Table 2). None of the group 1 patients had displaced metatarsal bones in the TMT joint region. After 3.7 years of follow-up, the median VAS-FA scores were 95.2, 94.4, 96.6, and 93.2 for overall, pain, function and other complaints, respectively (Fig. 2). From all grade 1 patients, 13 (59%) scored over 90 points in both the pain and function subscales of the VAS-FA, and 15 (68%) scored over 80 points. The overall score was over 90 points in 14 (64%) patients and over 80 in 17 (77%) patients. One patient underwent secondary surgery, an arthrodesis of the second TMT joint, after ten months of follow-up.

**Table 2:** Characteristics of the nonoperatively treated Lisfranc injuries classified by the Schepers and Rammelt classification

	*n*	Follow-up (years)	Number of TMT joints	Number of dislocated TMT joints	Medial column	Central column	Lateral column
		Mean	Median (range)	Median (range)	*n* (%)	*n* (%)	*n* (%)
All^a^	175	–	3 (1–5)	0 (0–4)	97 (55)	140 (80)	98 (56)
Included^b^	55	4.0	2 (1–5)	0 (0–1)	27 (49)	45 (82)	28 (51)
Grade^c^ 1	22	3.7	2 (1–3)	0 (0–0)	10 (45)	15 (68)	7 (32)
Grade^c^ 2	33	4.1	3 (1–5)	0 (0–0)	17 (52)	30 (91)	21 (64)

^a^All nonoperatively treated patients

^b^All nonoperatively treated patients who adequately completed the questionnaires

^c^Modified Schepers and Rammelt classification 1: avulsion fractures, 2: simple intra-articular fractures

Grade 2 injuries included 33 patients with simple non-displaced intra-articular fractures in the Lisfranc joint region. Patients had injuries affecting from 1 to 5 TMT joints (Table 2). None of the group 2 patients had displaced metatarsal bones in the TMT joint region. After 4 years of follow-up, the median VAS-FA scores were 94.2, 91.5, 97.0, and 92.2 for overall, pain, function and other complaints, respectively (Fig. 2). From all grade 2 patients, 17 (52%) scored over 90 points in both the pain and function subscales of the VAS-FA, and 21 (64%) scored over 80 points. The overall score was over 90 points in 21 (64%) patients and over 80 in 26 (79%) patients. One patient with an unsatisfactory result had been primarily missed, and nonoperative treatment was started 1 month after the injury. None of the patients with grade 2 injury underwent secondary surgery.

## Discussion

The main finding of our study is that non-displaced Lisfranc injuries affecting up to three TMT joints can be treated nonoperatively with good functional outcomes. The mean scores for patients without foot pathologies have been reported in the literature as follows: 94.5 for overall, 92.5 for pain, 95.4 for function, and 75.6 for other complaints [8]. In our study, half of the patients in all groups scored over 90 points in both the Pain and Function subscales and more than 60% scored over 90 points overall. Therefore, the results of this study show that most of the nonoperatively treated grade 1 and 2 patients in our study recovered close to the level of healthy patients during 2–6 years of follow-up. The distribution of the VAS-FA scores seemed to be similar between bony avulsions, simple intra-articular fractures. For example, the AO Foundation Surgery Reference [9] suggests that bony ligamentous injuries without displacement (grade 1) should be treated operatively. However, the results of this study show that patients with grade 1 injuries were successfully treated nonoperatively in our sample, yet we have to agree that the study population was rather small to draw any final conclusions. The secondary operation rate after 2–6 years of follow-up was low, since only 1 of the 55 patients had an arthrodesis of TMT II performed 10 months after the injury.

The limitations of our study are the retrospective nature and relatively low response rate (64%), which may cause a selection bias. Nevertheless, the clinical characteristics of all nonoperatively treated patients seem to be similar to the included sample, and the number of patients in this study is still larger than in previous studies [4, 5, 8, 34] investigating subtle Lisfranc injuries. Our study is also the first to evaluate the outcomes after nonoperatively treated Lisfranc injuries where the diagnosis of the injury was confirmed with CT imaging. One obvious limitation, which we chose already while planning the study, was not to use any clinical examination or imaging of the patients instead of evaluating the outcome with a validated PROM. This decision was taken because in previous studies, it has been clearly shown that radiological findings and the symptoms of postoperative osteoarthritis are not related [18, 20].

The largest previous study investigating the nonoperative treatment of Lisfranc injuries by Crates et al. [4] reported that up to 20 out of 36 patients underwent secondary surgery. Their nonoperative protocol was conducted with 6 weeks of short leg walking orthosis and weightbearing was allowed as tolerated. Moreover, the diagnosis was based on standard radiographs and, even though there were no findings in the radiographs, patients with remarkable clinical symptoms were included in the study. Additionally, the failure of nonoperative treatment was evaluated by a clinician, and further details of the reasons behind the conversion to operative treatment were not given. Due to these flaws, the results of the study can be questioned. The nonoperative protocol in our patients was more cautious in the sense of weightbearing than the one used by Crates et al. [4]. As our results suggest, the outcome may be better if the nonoperative protocol is started with non-weightbearing and the immobilization lasts for up to 6–10 weeks.

During recent years, only four retrospective studies of the conservative treatment of Lisfranc injury have been published [4, 5, 22, 34]. Two of the studies did not provide any criteria for nonoperative treatment, as patients with similar injuries were treated both operatively and nonoperatively [4, 5]. However, two of the studies [22, 34] used diastasis of < 2 mm between the medial cuneiform and the base of the second metatarsal bone as the threshold for nonoperative treatment. Both studies included seven nonoperatively treated patients [22, 34]. In all of these previous studies, the diagnosis of the injury was based on plain radiographs, and the evaluation of the outcomes was conducted without valid outcome measures [4, 5, 8, 22, 34]. However, when the diagnosis is based solely on plain radiographs, there is a possibility that injuries that would have benefited from more cautious treatment and also injuries that would not heal with nonoperative treatment, and, therefore, require operative treatment, are missed [26].

In the studies, the nonoperative treatment protocol used has varied from ‘none’ to 6 weeks of cast immobilization followed by 4 weeks of walking orthosis [4, 8, 20, 22, 34]. Only a few studies have suggested non-weightbearing during the immobilization [8, 22]. Even though there is no consensus on which patients should be treated nonoperatively, some authors have suggested that only stable injuries without any displacement should be treated nonoperatively [22]. Nevertheless, there are no successful techniques to determine whether an injury is stable or not, and, therefore, this statement needs to be considered carefully. Moreover, operative and adequately conducted nonoperative treatment have not previously been compared in a randomized controlled study setting.

Instability of the Lisfranc injury is suggested to be a sign of poor outcome [22]. However, diagnosing the instability of the Lisfranc injury still remains questionable [21, 27]. Multiple methods have been suggested to detect the instability, such as weightbearing radiographs and stress testing under fluoroscopy [22, 29]. Recent studies have, however, raised concerns about whether weightbearing radiographs have weak sensitivity and specificity to detect the instability [15, 26, 27]. In addition, the reference method for the evaluation, stress testing under fluoroscopy, has been shown to have low interobserver reliability, and, therefore, is not a practical way to detect the instability [21]. Hence, radiograph-based modalities and stress testing are not efficient, and computed tomography (CT) has been suggested to be the method of choice [10, 12, 26, 27].

The classification by Schepers and Rammelt [33] was developed to replace the previous radiograph-based classifications [13, 20, 22]. This modern classification divides the injuries based on fracture type (avulsion, simple, or comminuted) with a combination of affected columns, and, therefore, results in dozens of different classes. Although this classification seems to be the most reasonable classification system for Lisfranc injuries, it would benefit from further development and evaluation to guide the treatment of these injuries. Additionally, the inter- and intraobserver reliability of the classification should be evaluated in future studies to assess the reliability of the classification in everyday practice.

In conclusion, nonoperative treatment certainly has a role in the treatment of Lisfranc injuries, yet the clinical criteria for which injuries can be treated nonoperatively is still unknown. The results of our study supports the view that non-dislocated injuries, despite the number of affected columns or the type of the injury (avulsion or simple intra-articular fracture) of the Lisfranc joint, can be treated nonoperatively with 4–6 weeks non-weightbearing cast with good functional outcomes. There may also be some specific injury types among these non-dislocated injuries that may benefit from operative treatment, yet the evidence of these types of injury is scarce. In future, the preliminary findings of this study should be further evaluated in a prospective randomized controlled study setting, which would compare the nonoperative and operative treatment of mildly displaced injuries. More knowledge is also needed about the signs of instability of the midfoot injury to produce a classification that would better guide the treatment of our patients.
